# Recycling the recyclers: strategies for the immobilisation of a PET-degrading cutinase

**DOI:** 10.1007/s00449-025-03131-7

**Published:** 2025-02-02

**Authors:** Stefanie Fritzsche, Marcus Popp, Lukas Spälter, Natalie Bonakdar, Nicolas Vogel, Kathrin Castiglione

**Affiliations:** 1 Institute of Bioprocess Engineering, Department of Chemical and Biological Engineering, Friedrich-Alexander-Universität Erlangen-Nürnberg, Paul-Gordan-Straße 3, 91052 Erlangen, Germany; 2 Institute of Particle Technology, Department of Chemical and Biological Engineering, Friedrich-Alexander-Universität Erlangen-Nürnberg, Cauerstraße 4, 91058 Erlangen, Germany

**Keywords:** Plastic waste, Enzymatic depolymerisation, Polyethylene terephthalate, Stimulus-responsive polymers, Cross-linked enzyme aggregates, Kollicoat^®^

## Abstract

Enzymatic degradation of polyethylene terephthalate (PET) represents a sustainable approach to reducing plastic waste and protecting fossil resources. The cost efficiency of enzymatic PET degradation processes could be substantially improved by reusing the enzymes. However, conventional immobilisation strategies, such as binding to porous carriers, are challenging as the immobilised enzyme can only interact with the macromolecular solid PET substrate to a limited extent, thus reducing the degradation efficiency. To mitigate this challenge, this work compared different immobilisation strategies of the PET-degrading cutinase ICCG_DAQI_. Immobilisation approaches included enzyme fixation via linkers to carriers, the synthesis of cross-linked enzyme aggregates with different porosities, and immobilisation on stimulus-responsive polymers. The highest degradation efficiencies were obtained with the pH-responsive material Kollicoat^®^, where 80% of the initial enzyme activity could be recovered after immobilisation. Degradation of textile PET fibres by the cutinase-Kollicoat^®^ immobilisate was investigated in batch reactions on a 1 L-scale. In three consecutive reaction cycles, the product yield of the released terephthalic acid exceeded 97% in less than 14 h. Even in the fifth cycle, 78% of the maximum yield was achieved in the same reaction time. An advantage of this process is the efficient pH-dependent recovery of the immobilisate after the reaction, which integrates seamlessly into the terephthalic acid recovery by lowering the pH after hydrolysis. This integration therefore not only simplifies the downstream processing, but also provides a cost-effective and resource-efficient solution for both enzyme reuse and product separation after PET degradation, making it a promising approach for industrial application.

**Supplementary Information:**

The online version contains supplementary material available at 10.1007/s00449-025-03131-7.

## Background

The pollution of the Earth's ecosystems by plastics such as polyethylene terephthalate (PET) has become a severe environmental issue of the twenty-first century [1, 2]. With a global market volume of over 25 million tonnes in 2022, PET is widely used in plastic bottles and synthetic fibres due to its durability, ease of processing and gas barrier properties [3, 4]. In principle, it can be recycled through chemical, thermal, and mechanical processes [5]. In practice, however, these processes are often environmentally, economically and socially unsustainable, due to harsh and harmful reaction conditions and loss of plastic quality [4, 6]. In recent years, the focus has therefore shifted to the use of PET-degrading enzymes as natural biocatalysts. Various enzymes belonging to the class of esterases (EC 3.1), such as cutinases, lipases and PETases have been shown to hydrolyse PET into its monomeric components, terephthalic acid and ethylene glycol, under mild environmental conditions, providing a promising route for sustainable recycling [7–10]. By optimising biocatalysts and reaction conditions, high concentrations of up to 20% (w/v) PET can now be degraded in a manageable time, even less than 12 h [11, 12]. Further improvements in the economic and environmental efficiency of PET degradation could be achieved by recycling of the enzymes, given their stability in relation to the short processing times. Reusing the enzymes would reduce the cost of biocatalyst production and simplify the purification of the hydrolysis products terephthalic acid and ethylene glycol [13, 14]. These two products of enzymatic hydrolysis, especially terephthalic acid, can be used for the production of new plastics, such as PET and polybutylene terephthalate (PBT), whilst ethylene glycol, for instance, can also be used as a feedstock for microbial cell cultivation [15–17]. To facilitate separation and recovery, enzymes can be immobilised. In general, there are two types of enzyme immobilisation: physical entrapment and chemical fixation [18]. For macromolecular substrates such as PET, physical microencapsulation or membrane encapsulation does not provide sufficient mass transfer. In the field of chemical fixation, methods with and without the use of carrier materials can be considered. Carrier-free immobilisation systems are also applicable to enzymes converting macromolecular substrates and are a cost-effective immobilisation option, especially for extracellular enzymes [19–22]. Co-precipitation with starch can improve substrate accessibility and internal mass transfer by the production of cross-linked enzyme aggregates (CLEAs) with greater porosity and larger pores [23, 24]. Inorganic materials, natural or synthetic polymers, or magnetic nanoparticles can be used as carriers for chemical fixation [18, 25]. However, solid porous supports can hinder the efficiency of PET degradation, partly because most proteins are located within porous structures. As a result, only surface-exposed enzymes will initially interact with solid PET until the degradation products are small enough to diffuse into the pores. Additionally, even enzymes bound to the carrier surface might show poor interaction with the macromolecular PET substrate due to rigidification effects. This can lead to significant rate limitations compared to the enzymes used in soluble form [26, 27]. The flexibility of immobilised enzymes and thus the enzyme–substrate interaction when using solid supports can be increased using longer spacers between the solid carrier materials and the enzyme. For instance, attaching the enzyme to a longer aldehyde chain via a suitable functional group allows the enzyme to retain its molecular dynamics leading to more effective degradation [14, 28, 29]. In addition, smart carriers, such as water-soluble stimulus-responsive polymers, offer an elegant solution for macromolecular substrates [30]. These polymers undergo conformational changes in response to stimuli, such as temperature, pH, ionic strength, and light [31–33]. Biocatalysts can be adsorbed or covalently attached to these naturally derived or synthetic polymers [32]. Under suitable environmental conditions, the responsive polymer can then be converted to a soluble state, enhancing the flexibility of the bound PET-degrading enzymes and completely avoiding their localisation within porous structures during catalysis. This can facilitate substrate accessibility and therefore the catalytic reaction even with macromolecular and insoluble polymeric substrates such as PET. After the biocatalytic reaction, the enzyme–polymer conjugate can be easily separated by inducing a conformational change with an appropriate stimulus [31].

Cutinases from *Thermobifida fusca* and *Humicola insolens* (HiC) have already been immobilised with high yields and stabilities on various materials, including chitosan beads and other resins [34, 35]. PETase has also been successfully immobilised using magnetic nanoparticles [25]. However, immobilisation approaches are often used for hydrolysis of substrates with large surface areas, such as dispersible PET nanoparticles, or use a soluble surrogate substrate, such as nitrophenolic compounds, rather than the actual bulk substrate PET.

In this study, we evaluated different immobilisation methods of the PET-degrading cutinase ICCG_DAQI_, focussing on approaches that ensure good accessibility of immobilised enzymes to macromolecular PET: The methods used included ReliZyme™ solid carrier particles with flexible spacers, CLEAs and pCLEAs as carrier-free systems, and thermoresponsive and pH-responsive polymers that provide both good accessibility in their soluble state and efficient separation in their insoluble state. The cutinase is a leaf-branch compost cutinase (LCC) variant, initially thermostabilised by Tournier et al., whose specific activity towards PET was enhanced by the introduction of additional point mutations [17, 36]. As previously demonstrated, this PET-degrading enzyme can efficiently hydrolyse various polyester materials completely in less than 24 h [17, 36, 37]. In this work, the suitability of the different immobilisation strategies was investigated for two types of substrates. First, the general esterase activity was determined via a model degradation reaction, using the soluble, small substrate nitrophenylacetate (pNPA), which is hydrolysed to photometrically detectable nitrophenol. This assay is used to measure whether, and to what extent, the immobilised enzyme retains a general catalytic activity. Second, we investigated the actual targeted macromolecular PET degradation by incubating the immobilised enzymes with post-industrial PET fibres and measuring the yield of terephthalic acid as an indicator of PET degradation. The comparison of these two parameters allowed us to assess accessibility issues between the immobilised enzyme and the macromolecular PET substrate, ultimately with the aim of identifying the most efficient cutinase immobilisation method. Finally, we evaluated the recyclability of the immobilised enzyme by repetitive degradation experiments using the same, recovered enzyme immobilisates.

## Materials and methods

### Materials

Analytical grade salts, titrants and components for bacterial cell culture were obtained from Carl Roth GmbH (Karlsruhe, Germany). For the preparation of phosphate buffers, monopotassium phosphate and dipotassium phosphate were obtained from Carl Roth GmbH (Karlsruhe, Germany). Enzymes used for molecular cloning were from New England Biolabs (Ipswich, MA). p-Nitrophenyl acetate (pNPA), *N*-ethyl-*N*′-(3-dimethylaminopropyl)-carbodiimide hydrochloride (EDC) as well as N-hydroxysuccinimide (NHS) were purchased from Thermo Fisher Scientific (Waltham, MA). Dimethyl sulfoxide (DMSO), azobisisobutyronitrile (AIBN), acrylamide, and acrylonitrile were from Sigma Aldrich (St. Louis, MO). 25% aqueous glutaraldehyde (GA) solution as well as α-amylase (*Aspergillus oryzae*, 30 U mg^−1^) were purchased from Merck KGaA (Darmstadt, Germany). ReliZyme™ particles EP 403/S, HA 403/S and HFA 403/S were from Resindion S.r.l. (Binasco, Italy), whilst Kollicoat^®^ MAE 100 P was from Sigma Aldrich (St. Louis, MO). PET fibres (post-industrial textile fibres, 9% crystallinity [36]) were provided by Gneuss Kunststofftechnik (Bad Oeynhausen, Germany).

### Protein production and purification

The recombinant production of the cutinase ICCG_DAQI_ in *E. coli* BL21 (DE3) using autoinduction medium as well as the purification by immobilised metal affinity chromatography (IMAC) was performed as described before [36, 38, 39]. Protein purity was determined by PAGE with Coomassie staining, whilst quantitative determination of protein concentration was performed using a Pierce BCA Protein Assay Kit (Thermo Fisher Scientific, Waltham, MA). The purified protein was aliquoted in storage buffer (20 mM Tris, 300 mM NaCl, pH 8) and stored at − 80 °C until use.

### Esterase activity assay

General esterase activity was determined spectrophotometrically using nitrophenol substrates as previously described [36, 39]. Briefly, a substrate stock solution of pNPA was prepared in acetonitrile. The appropriately diluted enzyme solution was pre-incubated in the required volume of the reaction buffer (20 mM Tris, 10 mM NaCl, pH 8) in a 96-well microtiter plate at 37 °C according to the assay procedure already described in the literature [40]. After addition of the substrate, the release of p-nitrophenol was monitored immediately by measuring the absorbance at 405 nm for 10 min at 37 °C in a plate reader (Tecan, Männedorf, Switzerland) [40]. The p-nitrophenol concentration was quantified using the p-nitrophenol extinction coefficient (17.4 mM^−1^ cm^−1^) determined under reaction conditions [36]. The linear slope obtained was used to calculate the esterase activity, where one unit of esterase activity was defined as the release of 1 μmol p-nitrophenol per minute.

### Small- and large-scale PET hydrolysis

The hydrolysis of post-industrial PET substrate was performed either in miniaturised stirred-tank reactors (bioREACTOR 48, 2mag, Munich, Germany) or on a 1 L-scale in a stirred-tank reactor (Labfors 3, Infors HT, Bottmingen, Switzerland). Each miniaturised stirred-tank reactor (16 mL working volume), equipped with a magnetic inductive S-stirrer, contained 2 g L^−1^ of PET fibre substrate dispersed in phosphate buffer (100 mM, pH 9). Hydrolysis was carried out at 300 rpm and 70 °C for up to 24 h as previously described [36]. For large-scale PET hydrolysis (1 L working volume), a Labfors 3 system (Infors HT, Bottmingen, Switzerland) with a maximum working volume of 2 L and a 6-bladed Rushton turbine impeller was used [39]. Hydrolysis experiments were also performed in 100 mM phosphate buffer at 70 °C and pH 9 using 10 g L^−1^ PET fibres and stirring at 300 rpm. pH control was achieved by automatic addition of 1 M NaOH or 1 M HCl. For both reactor systems, enzymatic hydrolysis was initiated by the addition of free or immobilised enzyme. Samples were taken at regular intervals via sampling ports and analysed for the hydrolysis products terephthalic acid, ethylene glycol, mono-(2-hydroxyethyl)terephthalic acid (MHET) and bis-(2-hydroxyethyl)terephthalic acid (BHET) by high performance liquid chromatography (HPLC) as previously described [36]. The terephthalic acid yield was calculated as the ratio of its concentration measured by HPLC to the maximum possible concentration based on the initial PET concentration (maximum 1.73 g L^−1^ terephthalic acid for 2 g L^−1^ PET).

### Immobilisation on ReliZyme™ particles

For the immobilisation on ReliZyme™ particle types HFA and EP, no activation prior to immobilisation was necessary, whilst HA was first activated using GA. Activation was achieved by incubating the particle suspension (2% (w/v)) in water with GA (1.25 g L^−1^) for 2.5 h before the particles were separated and washed (10 min, 4500*g*). Both the non-activated HFA and EP particles and the activated HA particles (2% (w/v) in 100 mM phosphate buffer, pH 8) were incubated with the purified enzyme (2.2 mg_enzyme_ g_particles_^−1^) for up to 20 h with continuous shaking at room temperature. The enzyme-loaded particles were then isolated and washed thrice by centrifugation (10 min, 4500*g*) before 200 mg particles were used per hydrolysis batch in the miniaturised stirred-tank reactors with a working volume of 16 mL (100 mM phosphate buffer, pH 8) as described above. For the reference hydrolysis with free enzyme, the same amount of enzyme as used for immobilisation (0.44 mg per hydrolysis batch) was applied to ensure comparability.

### Synthesis of CLEAs

Different ammonium sulphate (AS) saturations for precipitation (50–80%) and GA concentrations for cross-linking (10–20 mM) were used for the preparation of CLEAs. Saturated AS stock solution and phosphate buffer (100 mM, pH 8) were added to glass flasks and stirred at 600 rpm (VARIOMAG POLY 15, Thermo Fisher Scientific, Waltham, MA) on ice. The enzyme solution was gradually added and precipitated for 1 h. Subsequently, cross-linking was initiated by the addition of GA, followed by incubation on ice for 4 h. After quenching the reaction by diluting with 100 mM lysine solution, the flasks were stored overnight at 4 °C. To separate unbound enzymes from the CLEAs, the samples were centrifuged at 4500*g* and 4 °C for 15 min. The supernatants were decanted and the remaining pellets were suspended in phosphate buffer and washed twice. The esterase activity of both the synthesised CLEAs and the washing supernatants was determined as described above, as well as the hydrolysis of PET in miniaturised stirred-tank reactors.

### CLEA modification by starch-coprecipitation (pCLEAs)

For the synthesis of pCLEAs with co-precipitation of starch, saturated AS stock solution and phosphate buffer (100 mM, pH 8) were added to glass flasks and stirred at 600 rpm, before 3% (w/v) starch was added simultaneously with the enzyme solution. The subsequent incubation for efficient precipitation and cross-linking steps followed those of a conventional CLEA synthesis, with the only difference being the addition of 30 U mL^−1^ of amylase to remove residual starch after the addition of lysine. Starch degradation was evaluated through the addition of a 0.05 M iodine solution, optically comparing the staining of samples co-precipitated with starch to those synthesised without starch [24].

### Reusability of CLEAs and pCLEAs

The stability of the general esterase activity of CLEAs and thus their recyclability for the ester hydrolysis reaction was investigated by repeating the pNPA assay as described above several times. After determination of the initial activities, the microtiter plate was centrifuged at 4000*g* and 4 °C for 5 min. The resulting supernatants were discarded, and the samples were resuspended in fresh reaction buffer, before the esterase activity was measured again. This procedure was repeated 10 times. To assess the recyclability of the CLEAs under PET hydrolysis process conditions, subsequent hydrolysis runs were performed as described above. After the first run, the reactor medium was centrifuged (4500*g*, 4 °C, 15 min), followed by washing and resuspension of the CLEA pellet in fresh hydrolysis buffer. A new hydrolysis run was initiated by the addition of PET fibres.

### Immobilisation on a thermoresponsive polymer

The thermoresponsive polymer poly(acrylamide-co-acrylonitrile) (p(AM-AN)) was synthesised by the polymerisation of acrylamide and acrylonitrile according to the literature [41]. Briefly, acrylamide and acrylonitrile were dissolved in DMSO in a ratio of 82:18 before the initiator AIBN was added. After 6 h at 65 °C under nitrogen atmosphere, the polymer was precipitated by the addition of methanol. After several rinses with methanol, the isolated polymer was dried at 70 °C for 24 h. To activate the polymer, 1% (v/v) GA was added to a 2% (w/v) polymer solution in deionised water and incubated with stirring for 6 h at 45 °C [41]. The activated polymer was separated, washed and then incubated with 1 mg mL^−1^ enzyme in phosphate buffer for 3 h at 45 °C. After this, the enzyme–polymer immobilisate was separated, washed twice and finally used for PET hydrolysis. To characterise the thermoresponsive behaviour of p(AM-AN), a 2% (w/v) polymer solution was incubated at temperatures between 5 and 50 °C and turbidity was assessed by measuring the extinction at 500 nm. The upper critical solution temperature (UCST) was determined using a sigmoidal least squares regression curve in GraphPad Prism. The inflexion point at 50% extinction corresponded to the UCST.

### Immobilisation on a pH-responsive polymer

A 2% (w/v) Kollicoat^®^ solution was prepared in deionised water by first increasing the pH to 11 with 2 M NaOH to ensure a complete dissolution and then reducing to pH 7 with 2 M HCl. For enzyme adsorption, 50 mL of the solution was mixed with 3 mg purified enzyme. If a covalent binding was intended, 0.6 g L^−1^ of NHS and EDC was added. After incubation for 3 h at 300 rpm and room temperature, the pH of the mixture was reduced to 4.5 with 3 M acetic acid and the precipitate was separated by centrifugation (400*g*, 10 min, 4 °C). The supernatant was discarded and the precipitate was washed twice with 0.01 M acetate buffer (pH 4.5). In the next step, the precipitate was redissolved in 50 mL phosphate buffer (pH 9), the pH was adjusted to 9 and the resulting polymer-enzyme solution was used for hydrolysis on a 1 L-scale in a Labfors 3 stirred-tank reactor as described above.

### Reusability of Kollicoat^®^

After hydrolysis, the medium was acidified to pH 4.8 with 3 M acetic acid. The resulting precipitate was separated by centrifugation (400*g*, 10 min, 4 °C), washed with acetate buffer (pH 4.5) and finally redissolved in 100 mM phosphate buffer (pH 9). The hydrolysis in the stirred-tank reactor was restarted according to the conditions described with the addition of fresh PET fibres. At the beginning and the end of each hydrolysis, the esterase activity (pNPA assay) and the hydrolysis products (HPLC) were analysed.

### Thermal and pH stability

Thermal stability was evaluated by incubating samples of free and immobilised enzyme in 100 mM phosphate buffer (pH 9) at 70 °C (Thermomixer, Eppendorf SE, Hamburg, Germany) for 14 days. General esterase activity was measured periodically and compared with the initial activity to determine the half-life of both free and immobilised enzyme at 70 °C. The pH stability was assessed by incubating the enzyme at pH values between 4 and 8 for up to 24 h at 70 °C und and then measuring the esterase activity in reaction buffer (20 mM Tris, 10 mM NaCl, pH 8).

### Statistical analysis

A two-sided *t*-test for independent samples (Excel) with a significance level of *α* ≤ 0.05 was used to determine whether the differences between individual results were statistically significant.

## Results

### ReliZyme™ as solid carrier particles

**Fig. 1:**
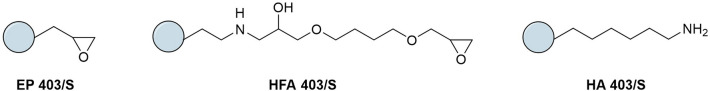
Overview of the functional groups of the carrier materials ReliZyme™ EP 403/S, HFA 403/S and HA 403/S. Figure created with ChemDraw Professional based on [29]

As the first immobilisation approach, a classical carrier fixation using synthetic polymer particles as support was investigated. For this purpose, methacrylic acid-based ReliZyme™ particles with different surface functionalisation and spacer lengths were used. HFA 403/S and EP 403/S differ in the length and structure of their alkyl spacers. Both have terminal epoxy groups that allow direct, covalent immobilisation of the cutinase via lysine residues on the protein surface (Fig. 1). In contrast, HA 403/S requires activation of its hexamethylamino spacer prior to immobilisation (Fig. 1). Incubation with the bifunctional GA leads to the formation of aldehyde groups which allow binding to the amino groups of the enzyme [42].

**Fig. 2:**
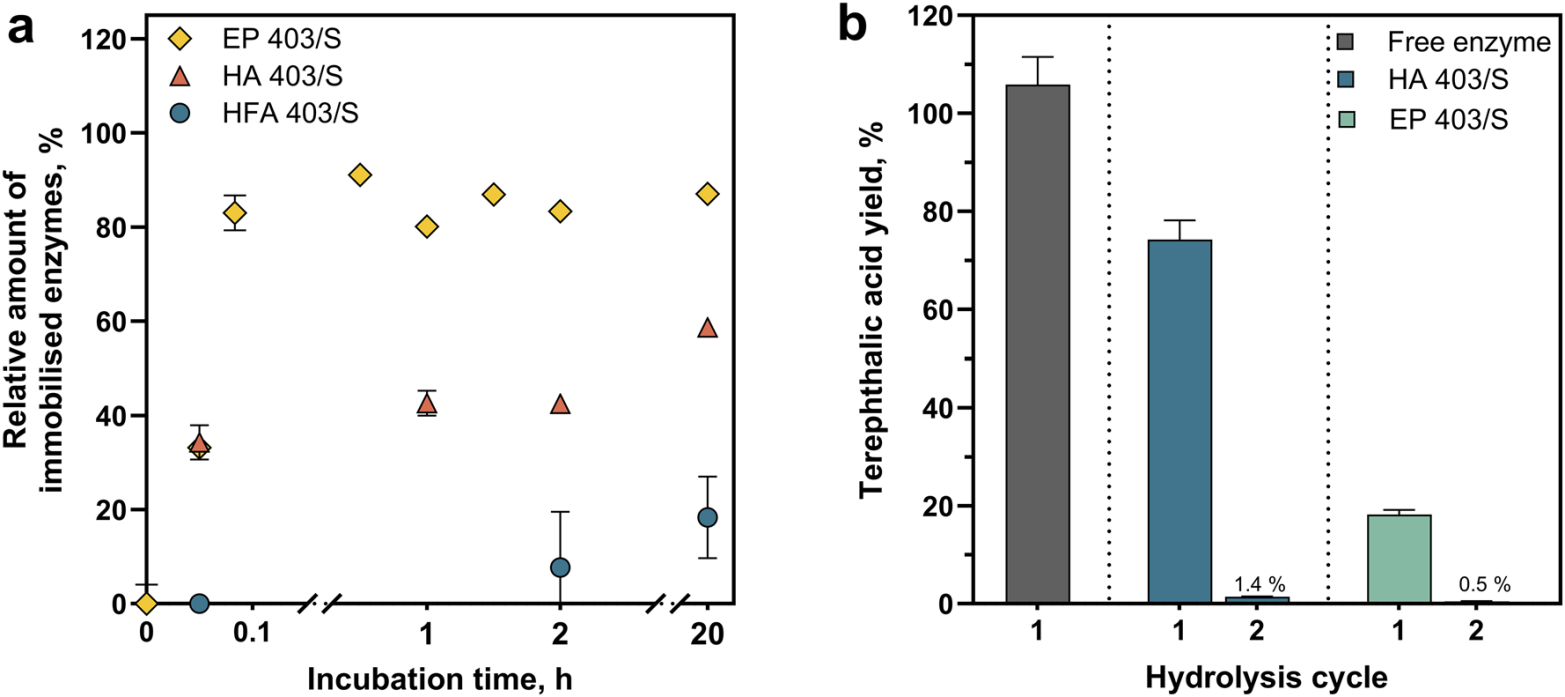
PET hydrolysis using cutinase immobilised onto solid ReliZyme™ carriers. **a** Relative amount of immobilised enzyme during the incubation of cutinase ICCG_DAQI_ with ReliZyme™ particles HFA 403/S, HA 403/S and EP 403/S. Each data point represents the mean of three pNPA-measurements. **b** Terephthalic acid yields in relation to the theoretical maximum during the hydrolysis of textile PET fibres with ICCG_DAQI_ as free enzyme (0.44 mg per reactor) or immobilised on different ReliZyme™ carriers (HA 403/S and EP 403/S, per reactor: use of 0.44 mg_enzyme_ for immobilisation) on a 16 mL-scale. Using the immobilised enzyme, the particles were separated after hydrolysis and used to hydrolyse fresh PET fibres

To assess the general suitability of cutinase immobilisation, the relative amount of immobilised enzymes was determined by measuring the residual esterase activity in the supernatants during the incubation of the cutinase ICCG_DAQI_ with the particles. Only low immobilisation efficiencies of ICCG_DAQI_ were achieved with HFA 403/S (< 20% immobilised enzymes) (Fig. 2a). This low efficiency may be due to differences in the length and structure of the alkyl chains between HFA 403/S and EP 403/S (Fig. 1), which appear to limit efficient immobilisation for HFA 403/S, as has been observed for other enzymes [43]. In addition, the reduced stability of HFA 403/S at temperatures above 10 °C and its short storage stability [44] likely further compromise its suitability for cutinase immobilisation under the conditions used in this study. In contrast, for HA 403/S and EP 403/S particles, the amount of immobilised cutinase was 60% and 80%, respectively after 20 h incubation.

These two types of particles, functionalised with epoxy and GA-activated amino groups, were therefore used for the hydrolysis of post-industrial PET fibres. In the first hydrolysis cycle, a release of terephthalic acid, indicating the successful degradation of PET into its monomer, was observed for both cutinase-ReliZyme™ immobilisates with efficiencies of 74% for HA 403/S particles and 18% for EP 403/S particles (Fig. 2b). The degradation efficiency was less effective compared to that of the free enzyme, which completely hydrolysed all available PET (Fig. 2 b). In addition, the yield of terephthalic acid decreased significantly in the second cycle to less than 2% for both cutinase particle immobilisates. The terephthalic acid equivalents, i.e. the sum of all degradation products including the intermediate products MHET and BHET, showed the same trends (Table S1). The results therefore show that although cutinase could be immobilised on solid particles via appropriate linker groups, it was not possible to achieve efficient PET hydrolysis, particularly with recycled immobilised enzymes.

### Carrier-free immobilisation using CLEAs

CLEAs are a carrier-free immobilisation method where proteins are first precipitated to form aggregates, which are then stabilised by cross-linking with a cross-linking agent [22]. For the first precipitation step, the two most commonly used precipitants, AS and PEG, were compared for their suitability to precipitate the cutinase ICCG_DAQI_ (Fig. S1). PEG showed poor esterase activity recovery (< 10%) at the PEG saturations tested (30, 50 and 70%), whereas AS yielded the best activity recovery of 70% at a saturation of 70%.

**Fig. 3:**
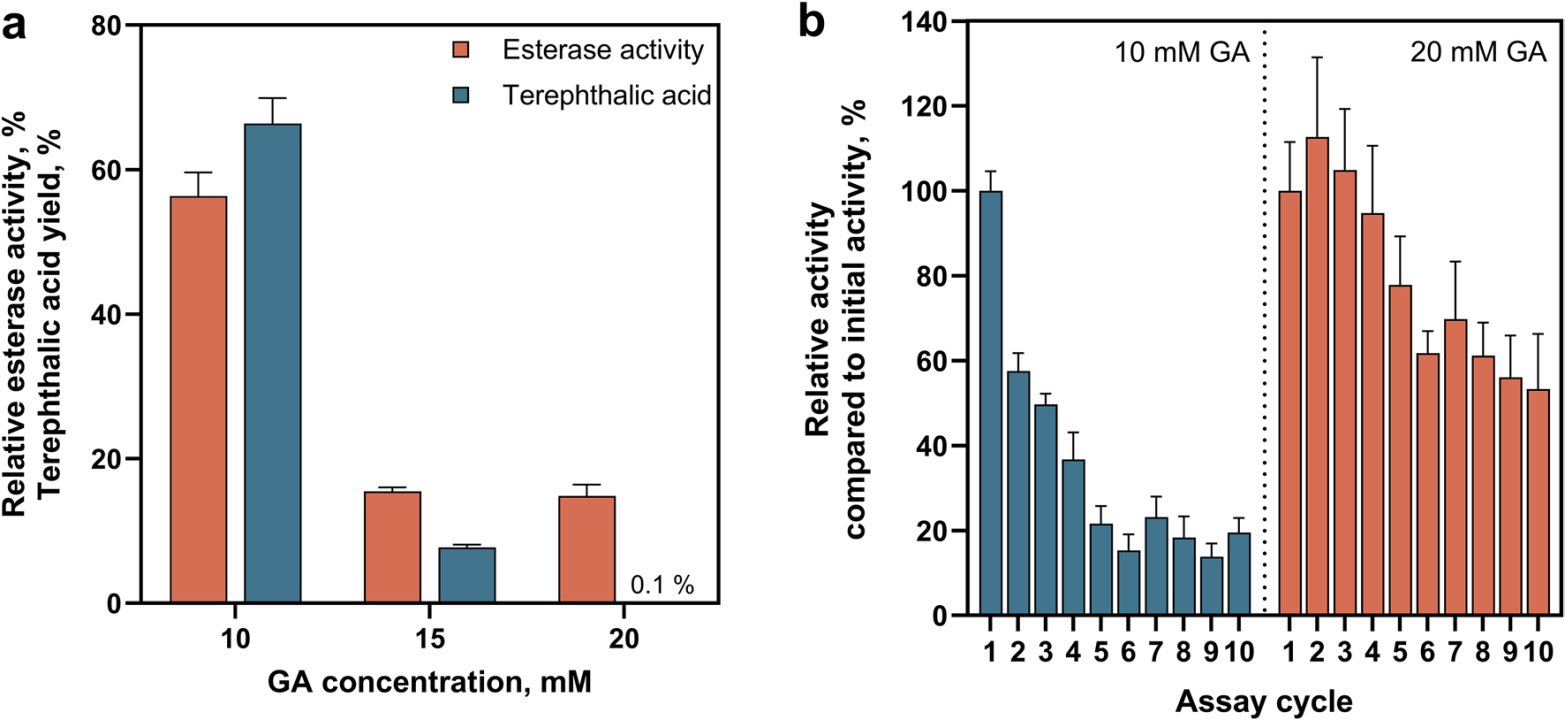
PET hydrolysis using CLEAs. **a** Terephthalic acid yield and relative esterase activity for different GA concentrations used in CLEA synthesis with constant AS saturation. PET fibres were hydrolysed in miniaturised stirred-tank reactors. The relative esterase activities were compared to the initial free esterase activity used for CLEA production (pNPA assay, *n* = 3). Terephthalic acid yields have a relative error of 5.3%, taking into account errors in weighing small amounts of fibre, sample preparation and HPLC measurement. **b** Relative catalytic esterase activity (pNPA assay) of CLEAs compared to their initial activity. The initial esterase activity of less cross-linked CLEAs (10 mM GA) was 69% of the activity of the free enzyme used to prepare the CLEAs, and that of the more cross-linked CLEAs (20 mM GA) was 24% of the activity of the free enzyme used. After each cycle, the centrifuged supernatant was discarded, the resulting pellet was washed, and fresh pNPA substrate was added to start the reaction. Each data point represents the mean ± standard deviation of three esterase measurements

In the second step of CLEA production, the protein aggregates formed after precipitation are stabilised with a cross-linking agent, the concentration of which is crucial for both CLEA stability and catalytic activity. In this work, GA was used as a cross-linker with concentrations between 10 and 20 mM. With increasing GA concentration, both the immobilisation efficiency, measured as the relative esterase activity of the CLEAs compared to the initial activity, and the terephthalic acid yield in PET fibre degradation decreased (Fig. 3a). We hypothesise that this decrease is caused by mass transfer limitations and reduced substrate accessibility due to denser cross-linking resulting in a more compact CLEA structure. The observed differences between general esterase activity (small pNPA substrate) and terephthalic acid yield (macromolecular PET substrate) support this hypothesis: at higher cross-linking densities, the cutinase can still hydrolyse small substrates, but PET cannot be enzymatically degraded due to accessibility limitations. When accessing the reusability, the weakly cross-linked CLEAs (10 mM GA) showed a faster decrease in esterase activity compared to the more cross-linked samples, with less than 20% activity measured after five recycling cycles (Fig. 3b). CLEAs with higher cross-linking density (20 mM GA) had a lower initial esterase activity compared to the free enzyme (100% esterase activity). However, amongst the GA concentrations tested, it showed the smallest loss of activity over the recycling cycles in the model reaction, with more than 50% of the initial activity measured after ten times of recycling (Fig. 3b). Stronger cross-linking appears to form more stable, compact aggregates, reducing enzyme loss and making CLEAs easier to separate and recycle, thereby preserving activity over cycles.

Unfortunately, the reduced stability of less cross-linked CLEAs was also observed under real process conditions (PET fibres, stirred-tank reactor, 70 °C, 22 h), when the CLEAs were isolated after the first PET hydrolysis, purified and reused for a second hydrolysis. Weakly cross-linked CLEAs (10 mM) achieved moderate PET degradation in the first cycle, yielding 67% terephthalic acid (Fig. 3a). However, in the second cycle, the degradation achieved was minimal, with only 5% terephthalic acid yield (Fig. S2). The observation that reusability of the CLEAs and high hydrolysis activity with solid PET as the substrate were mutually exclusive could be due to the poor substrate accessibility at higher degrees of cross-linking. Nevertheless, higher cross-linking seems necessary to maintain stability and hence reusability of CLEAs under process conditions of 70 °C for more than 20 h.

### Carrier-free immobilisation using pCLEAs

**Fig. 4:**
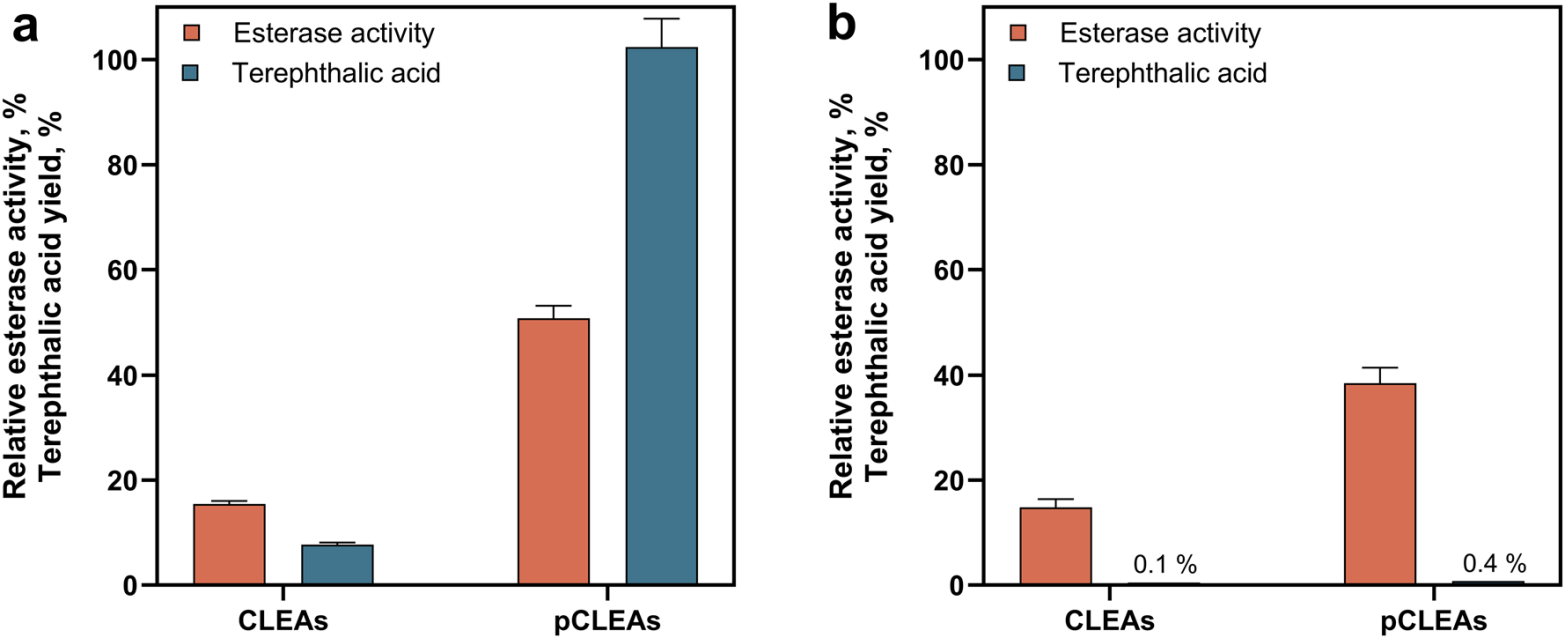
Comparison of CLEAs and pCLEAs for cutinase immobilisation. Shown are both the terephthalic acid yield and relative esterase activity for CLEAs and pCLEAs with 15 mM (**a**) and 20 mM (**b**) cross-linker concentration. PET fibres were hydrolysed in miniaturised stirred-tank reactors. Terephthalic acid yields have a relative error of 5.3%, taking into account errors in weighing small amounts of fibre, sample preparation and HPLC measurement. The relative esterase activities were compared to the initial free esterase activity used for CLEA production (pNPA assay, *n* = 3)

One concept to overcome the accessibility obstacle is to increase the porosity of CLEAs at higher cross-linking through the co-precipitation of starch and enzymatic removal of the starch after cross-linking [23, 24]. Such porous CLEAs (pCLEAs) are advantageous because they can reduce mass transfer limitations and accessibility issues for macromolecular enzymatic substrates, thereby enhancing catalytic efficiency [24]. For the synthesis of pCLEAs, starch was precipitated simultaneously with the enzyme using AS as precipitating agent. Amylase was added to remove the starch after cross-linking with GA to create the porous structure [24]. After synthesis, we first assessed the effect of increased porosity via the general esterase activity using the model degradation reaction with the small and soluble substrate pNPA for the highly cross-linked stable CLEAs (20 mM) and an intermediate cross-linker density (15 mM). These pCLEAs with medium to high cross-linker concentrations resulted in a substantial increase in general esterase activity, suggesting that, at least for the smaller substrate pNPA, there was indeed less diffusion limitation within pCLEAs compared to conventional CLEAs (Fig. 4). In particular, for pCLEAs with 15 mM GA, esterase activity could be improved over threefold via the porosity added by starch co-precipitation (Fig. 4a). Similarly, an enhanced catalytic activity of the pCLEAs compared to the non-porous CLEAs was also found during PET degradation, where successful PET fibre hydrolysis (102 ± 5% terephthalic acid yield) was observed for pCLEAs cross-linked with 15 mM GA (Fig. 4a). Although pCLEAs with 20 mM GA showed a fourfold increase in general esterase activity in the model reaction, no substantial release of terephthalic acid (< 10%) was measured when using PET fibres as substrates (Fig. 4b). The observed difference between general esterase activity and PET hydrolysis can be attributed to the different substrates (pNPA versus PET) and the resulting different effects of cross-linking on mass transport and substrate accessibility to the enzymes. Soluble substrates can diffuse into the pores, and thus increase the overall activity. In contrast, the insoluble, macroscopic fibres only provide limited contact areas, thus reducing the overall efficiency of the degradation process.

Although pCLEAs with intermediate cross-linking density (15 mM) showed promising results for PET hydrolysis (Fig. 4a), their recyclability appeared to be limited. In the second hydrolysis cycle, esterase activity dropped below 20% and terephthalic acid release was minimal (3% yield), probably due to reduced stability from higher porosity despite the increased cross-linking. In conclusion, neither CLEAs nor pCLEAs showed the desired combination of catalytic activity and recyclability.

### Immobilisation on soluble carriers

**Fig. 5:**
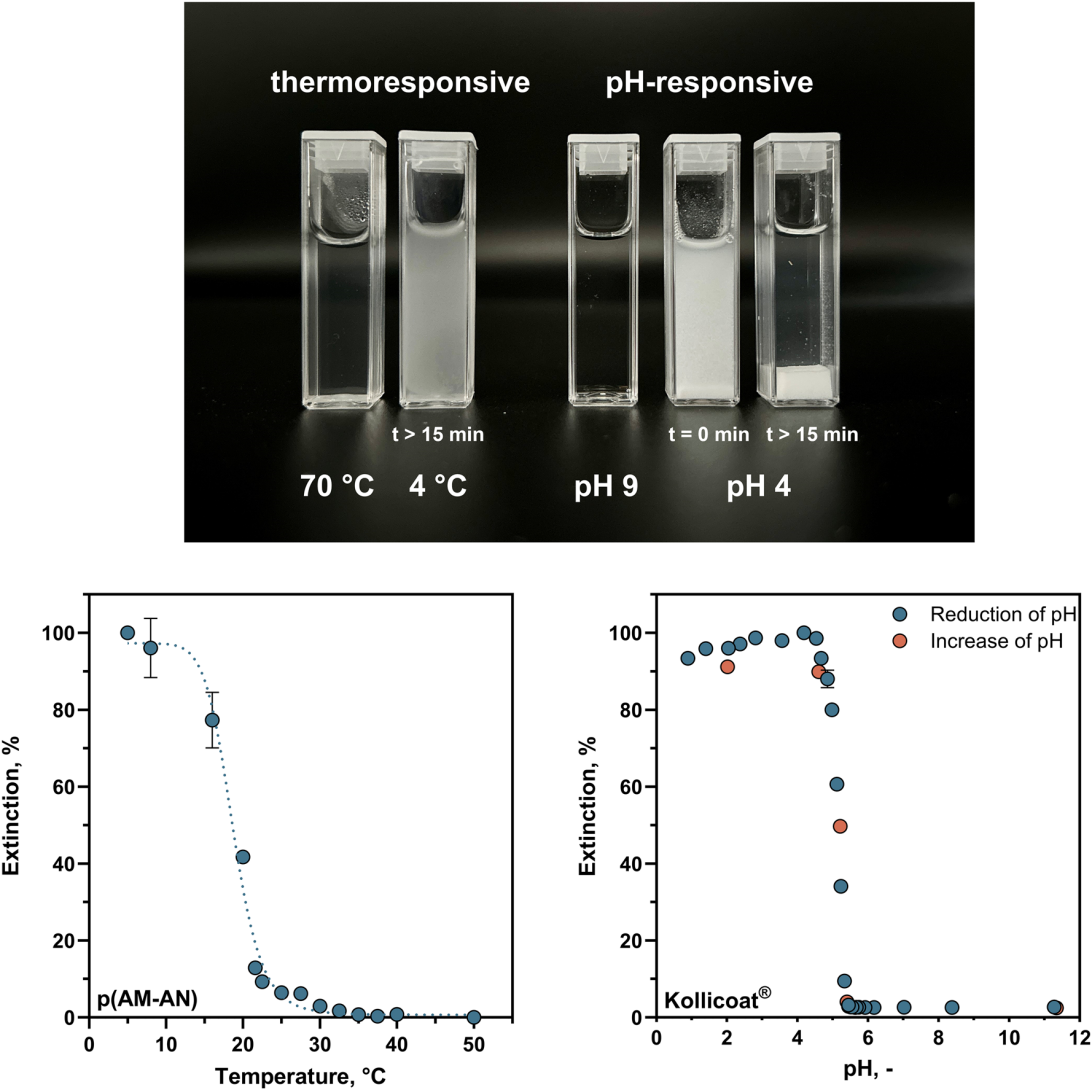
Thermoresponsive polymer (p(AM-AN)) and pH-responsive polymer (Kollicoat^®^) under their respective hydrolysis conditions (70 °C, pH 9) and separation conditions (4 °C, pH 4). For the pH-responsive polymer, a clear collapse of the precipitated polymer by sedimentation was observed after a waiting period of 15 min. Also shown is the extinction at a wavelength of 500 nm of a Kollicoat^®^ solution (2% (w/v)) in the pH range from 1 to 12 and a p(AM-AN) solution (2% (w/v)) in the temperature range of 5–50 °C. Each value represents the mean value, normalised to the maximum extinction of three technical replicates ± standard deviation. The UCST of p(AM-AN) was determined at 50% extinction using a sigmoidal least squares regression curve (*R*^2^ = 0.99, dotted line) in GraphPad Prism

pH- and thermoresponsive polymers may offer a promising approach to enzyme immobilisation as they exhibit different conformations depending on process conditions. In particular, the polymers can be highly swollen or collapsed depending on the external conditions. When used as a carrier for enzyme immobilisation, these conformational changes provide a way to combine both efficient separation properties and accessibility to solid substrates [30, 45, 46]. At PET hydrolysis conditions of 70 °C and pH 9, the polymers remain soluble, ensuring optimal substrate accessibility for the immobilised enzyme. After hydrolysis and cooling, thermoresponsive polymers undergo a volume phase transition, collapsing into a coiled state and allowing easy separation of the immobilised enzyme. Similarly, when the pH is lowered for terephthalic acid recovery [47], a pH-responsive polymer can become insoluble around pH 5, allowing the enzyme-polymer complex to be removed before lowering the pH further below 4 to separate the terephthalic acid.

p(AM-AN) was selected as a thermoresponsive polymer [41]. A 2% (w/v) p(AM-AN) solution showed thermoresponsive behaviour as the transparent solution of the polymer at 70 °C became turbid when cooled to 4 °C, indicating that the polymer precipitated (Fig. 5). We assessed this precipitation by temperature-dependent extinction measurements at 500 nm and determined an UCST (50% extinction) of 19 °C (Fig. 5). After activation of the amine groups of the polymer with GA, the resulting aldehyde groups reacted with the amine groups of cutinase to form a cutinase-p(AM-AN) immobilisate. When this immobilisate was used for PET fibre hydrolysis, over 80% terephthalic acid was obtained in the first cycle (Fig. S3). However, when the cutinase-p(AM-AN) immobilisate was separated after hydrolysis and reused for a second hydrolysis cycle, the immobilisate could not be well separated from the aqueous phase, ultimately resulting in an inefficient yield of only 1% in the second hydrolysis cycle (Fig. S3).

pH-responsive polymers exhibit a swelling or collapsing behaviour in response to pH changes, affecting electrostatic interactions and consequently the hydrophobicity of the polymer chain [48]. This swelling-collapse-transition could be clearly seen for the pH-responsive polymer Kollicoat^®^ (Fig. 5). This methacrylic acid–ethyl acrylate copolymer has two different solubility states, depending on the pH value. At a pH above 5.5, the carboxyl groups in the polymer are deprotonated and form a water-soluble polyelectrolyte, leading to complete dissolution in water. Conversely, at pH values below 5.5, the solubility of the polymer decreases due to protonation, leading to reversible precipitation [49, 50]. We investigated the pH-responsiveness of Kollicoat^®^ by measuring the extinction at 500 nm of a 2% (w/v) solution over a pH range of 1 to 12. A significant change in extinction was observed between pH 5 and 6, indicating the swelling–collapse–transition of the polymer (Fig. 5). Below pH 5, the polymer remained completely undissolved (extinction > 90%), whereas above pH 6, it was completely dissolved with extinction values below 5% (Fig. 5). A pH of 4.8 was therefore chosen for the precipitation process of Kollicoat^®^ in this work, which is in line with values reported in the literature for structurally identical Eudragit^®^ [46, 51, 52].

**Fig. 6:**
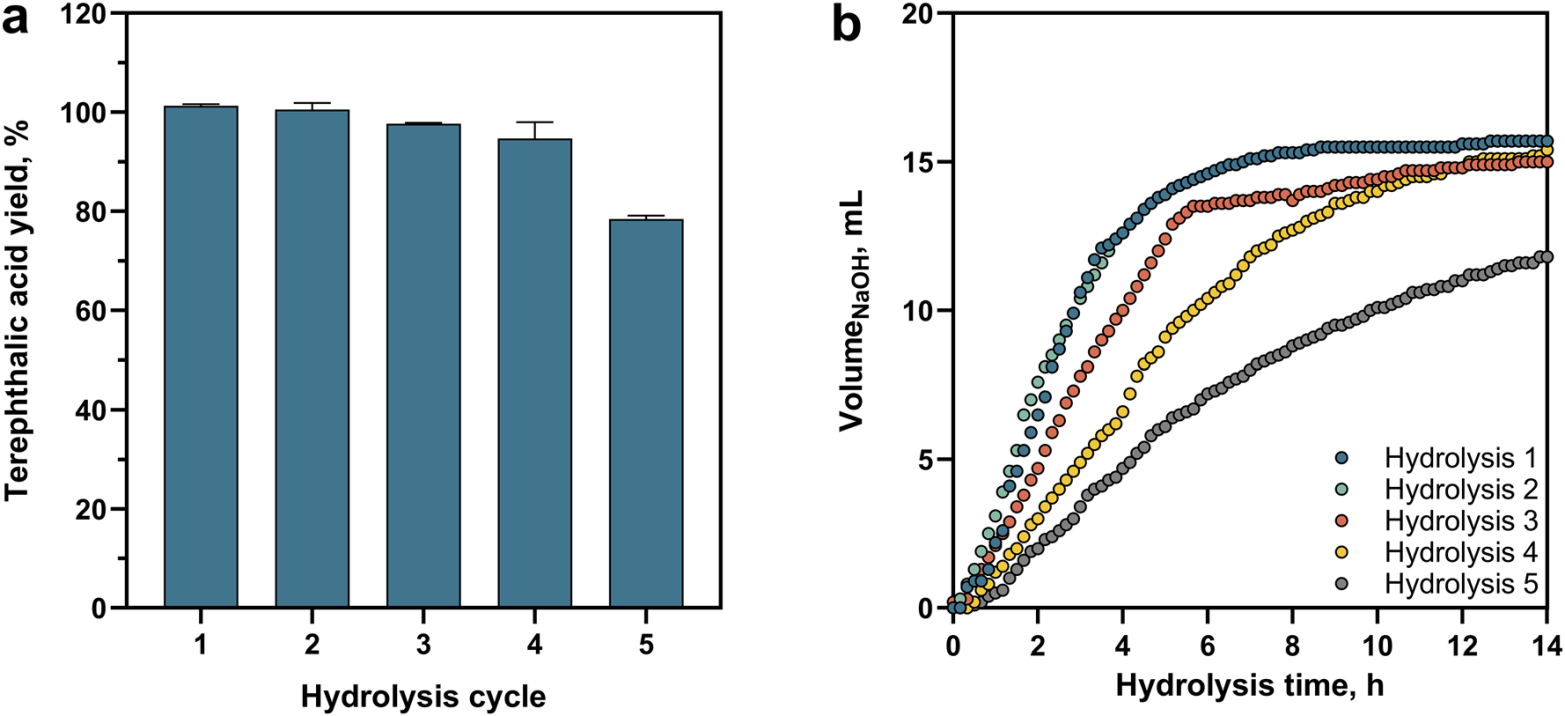
PET hydrolysis with cutinase-Kollicoat^®^ immobilisates. **a** Terephthalic acid yields in relation to the theoretical maximum after the hydrolysis of textile PET fibres with cutinase ICCG_DAQI_ immobilised on Kollicoat^®^ on a 1 L-scale in a stirred-tank reactor. The maximum release of the intermediate products MHET and BHET was less than 3%. After each of the five hydrolysis cycles, Kollicoat^®^ was precipitated by alteration of the pH, washed and reused for the hydrolysis of fresh PET fibres. **b** Automatic NaOH addition profiles for maintaining a constant pH despite the terephthalic acid release during PET hydrolysis on a 1 L-scale in a stirred-tank reactor, plotted over 14 h for the five consecutive hydrolysis cycles

When 3 mg cutinase ICCG_DAQI_ was immobilised with 2% (w/v) Kollicoat^®^, via the addition of NHS and EDC as coupling agents at pH 7, less than 1% of the initial esterase activity was detected in the supernatant and subsequent washes (Fig. S4). The immobilisation efficiency, measured as the relative esterase activity of the resulting immobilisate compared to the free enzyme activity was 81%, indicating sufficient loading of the polymers with active enzyme. The reduced specific activity of the recovered cutinase is probably due to conformational changes of the cutinase or steric hindrance as reported for other enzymes in the literature [46, 51]. The cutinase-Kollicoat^®^ immobilisate showed successful degradation of PET fibres on a 1 L-scale in a stirred-tank reactor, with terephthalic acid yields of over 97% in less than 14 h for the first three hydrolysis cycles. This almost complete conversion was also achieved with recycled cutinase-Kollicoat^®^ immobilisates, which could be precipitated from the reaction solution by changing the pH to 4.8. Even in the fifth recycling cycle, the yield of terephthalic acid remained at 78% (Fig. 6a). This reduction of PET yield after several hydrolysis cycles on a 1 L-scale is reflected by the decrease of the general esterase activity over the cycles (Fig. S5a). Both thermal inactivation of cutinase and incomplete recovery of the immobilisate during recycling contribute to the observed decrease in activity over cycles. This is indicated by the loss of esterase activity observed during the hydrolysis cycles, as well as the sharp decline during the recycling step (Fig. S5b). Further optimisation of the immobilisate separation process could potentially minimise the loss of enzymatic activity during recycling. The observation of terephthalic acid yields was also observed in the NaOH consumption curves. During the hydrolysis, NaOH was automatically dosed to maintain a stable pH of 9 in the stirred-tank reactor to neutralise the formation of terephthalic acid from the PET hydrolysis. Whilst the curves during the first and second hydrolysis cycles showed no noticeable differences, from the third hydrolysis cycle onwards, a reduced NaOH addition rate and consequently a slower hydrolysis rate was observed (Fig. 6b). By the fifth hydrolysis cycle, less NaOH was needed to maintain a pH of 9 in the 1 L stirred-tank reactor, corroborating the reduced terephthalic acid yield. Nevertheless, the cutinase-Kollicoat^®^ immobilisates showed substantially improved recyclability compared to all other strategies.

As also non-covalent immobilisation on responsive polymers is described in the literature, the immobilisation of cutinase without addition of EDC for covalent binding was tested [52, 53]. This physisorbed cutinase-Kollicoat^®^ immobilisate also showed effective hydrolysis of post-industrial PET fibres. Although the terephthalic acid yield decreased more rapidly after the initial recycling processes compared to the covalently immobilised enzyme shown in Fig. 6a, a final terephthalic acid yield of 79% was achieved after five consecutive times of hydrolysis and recycling (Fig. S6). In both cases, the cutinase-Kollicoat^®^ immobilisate was easily separated by lowering the pH to 4.8. At this pH, the responsive polymer Kollicoat^®^ was efficiently precipitated whilst the hydrolysis product terephthalic acid remained in solution. Further lowering of the pH to 2.5 allowed the complete removal of terephthalic acid from the hydrolysis medium. Therefore, this approach allows the isolation of the cutinase-Kollicoat^®^ immobilisate separate from the terephthalic acid. Based on HPLC measurements before and after the precipitation procedure, over 90% of the released terephthalic acid was recovered as the final isolate after separation of the immobilised cutinase.

As described above, the pH is lowered to 4.8 during the precipitation step to recover the cutinase-Kollicoat^®^ immobilisates. To evaluate the effect of this low pH on the stability of the immobilisate and to determine whether immobilisation has a stabilising effect on the enzyme, both the free enzyme and the immobilisate are incubated at different pH values at 70 °C for up to 24 h and then esterase activity was measured using the pNPA assay at pH 8. Compared to the free enzyme, all immobilised samples retained higher esterase activity at all pH values (Table S2), indicating improved pH and thermal cutinase stability when immobilised on Kollicoat^®^. Stability tests showed no significant loss of activity of the immobilisate after 30 min and 6 h at pH 4 (*p* ≤ 0.43 and *p* ≤ 0.14). Therefore, no adverse effects on enzyme activity or stability are expected when the pH is lowered to recover Kollicoat^®^ between cycles.

## Discussion

### Reusing enzymes with solid carriers

The immobilisation of the cutinase ICCG_DAQI_ on methacrylic acid-based particles was strongly influenced by the functional groups present on the particles, as has also been observed in other studies [43, 54]. In this work, the highest immobilisation efficiencies of over 80% were achieved using ReliZyme™ EP 403/S (Fig. 2a), which is comparable to other studies (50 to 100%, depending on the target enzyme and loading conditions) [54, 55]. Despite the high immobilisation efficiencies, the use of these loaded particles for PET hydrolysis resulted in low product yields with the solid substrate PET especially after a recycling step (Fig. 2b), indicating problems with substrate accessibility and steric hindrance. Similarly, Lu et al*.* [56] showed good general activity by immobilising ICCG on larger resin particles (200 µm diameter), but observed poor PET degradation, likely due to accessibility issues. In contrast, immobilisation on diatomaceous earth (20 µm diameter) enabled successful PET degradation in a rotating packed bed reactor when combined with β-cyclodextrin to mitigate shear stress. However, this approach also showed limitations in terms of reusability [56]. Therefore, although solid supports have been shown to effectively immobilise enzymes for the conversion of macromolecular substrates [25, 56–58], particle size and porosity have a substantial effect on mass transfer and efficiency. In this study, porous particles were selected for PET hydrolysis due to their potential to facilitate the hydrolysis of intermediates, such as MHET and BHET, by enzymes immobilised within the pores. This mechanism could complement the action of surface-bound enzymes that degrade macromolecular PET. However, further investigation of carrier particles with different structures, materials, diameters and porosities was not pursued within this study.

### Carrier-free immobilisation

Carrier-free immobilisation strategies like CLEAs can be a suitable option for the hydrolysis of solid substrates as has been demonstrated for enzymes involved in the degradation of lignocellulosic biomass or bioplastics, such as cellulases and poly-3-hydroxybutyrate depolymerase [19, 20, 22, 59]. However, finding the sweet spot between maintaining catalytic activity and ensuring recyclability is generally a particular challenge with CLEAs, especially when a bulky substrate is to be hydrolysed. The concentration of the cross-linking agent in the CLEA synthesis is critical as it controls covalent binding between enzymes, which influences not only aggregate stability, but might also cause diffusion problems and reduced substrate accessibility [21, 60, 61]. For cutinase-based CLEAs, a combination of high catalytic activity of the cutinase towards PET substrates with recyclability of the immobilisate could not be identified (Fig. 3). pCLEAs were then synthesised to address these shortcomings as their larger pore structure is likely to reduce mass transfer limitations and an increase in substrate accessibility. This increased porosity shows an improved esterase activity of the pCLEAs compared to CLEAs (Fig. 4), but also resulted in decreased stability and recyclability. This is consistent with previous reports of pCLEAs for other enzymes, which also reported enhanced accessibility and activity compared to CLEAs [21, 23, 24]. However, the trade-off between high degradation efficiency and reusability of CLEAs shown in this work was also evident for other CLEA approaches. For example, CLEAs based on *Ideonella sakaiensis* PETase and amylopectin as a macromolecular cross-linker for improved substrate accessibility effectively degraded macromolecular PET substrate films achieving complete degradation (100% product yield) after 32 h, though recycling efficiency was limited. After 8 h of hydrolysis, CLEAs could be reused three times, yet product yields remained below 40% in this shorter time frame [62]. Additional strategies, such as co-precipitation with bovine serum albumin or alternative cross-linkers like polyethyleneimine and oxidised dextran, may further improve both accessibility and stability [62–65].

### Immobilisation on stimulus-responsive polymers

Using the thermoresponsive polymer p(AM-AN) for immobilisation of PET-degrading enzymes was not successful as the precipitate could not be efficiently separated at temperatures below UCST in this work as no collapse into a sufficiently dense aggregate occurred (Fig. 5) [41]. Using the pH-responsive polymer Kollicoat^®^ MAE 100 P [49], a cutinase immobilisation efficiency of over 80% could be achieved. Notably, less than 1% active cutinase loss was detected in the incubation or wash supernatants (Fig. S4), which indicates a reduced specific activity of the bound enzyme. Similar effects have been also reported in the literature, with differences of up to 30% between enzyme binding and activity measurements [51, 66, 67]. Possible reasons for this decreased activity include steric hindrance of the enzyme's active site or substrate-binding groove by the support material or loss of enzyme flexibility as shown by kinetic analysis and reduced substrate affinity for enzymes immobilised on structurally similar Eudragit^®^ [45, 46].

The literature describes both physisorption and covalent linkage for successful immobilisation of enzymes on pH-responsive polymers such as Eudragit^®^ or Kollicoat^®^ [45, 46, 52, 66, 68–72]. In this work, both options were investigated and NHS was used in addition to EDC to increase binding efficiency by stabilising the intermediate product of bond formation [73]. Complete PET hydrolysis was achieved with and without EDC, and although immobilisation with EDC showed a slower decline in yield and esterase activity over the cycles, both methods reached similar degradation yields by the fifth cycle for PET hydrolysis on a 1 L-scale (Fig. 6, Fig. S6). As there was no significant difference between immobilisation with and without EDC in this study, it remains unclear whether the addition of EDC and NHS led to the formation of covalent bonds. However, the slight differences in thermal stability between immobilisation with and without EDC (Table S2) suggest that covalent bonds have formed. In the literature, both increased thermal and pH stabilisation through covalent bonding and adsorption have been observed [46, 52, 66, 68].

Although many different enzymes have been immobilised using a pH-responsive polymer before [45, 46, 52, 66, 68–72], to our knowledge, there have been no reports of successful immobilisation of PET-degrading enzymes on pH-responsive polymers such as Kollicoat^®^. From an economic point of view, this carrier material is well suited as it is already produced on a large-scale for the pharmaceutical industry as a tablet coating material [49]. In this work, only 0.1% (w/v) final concentration of Kollicoat^®^ was used during PET hydrolysis, making the cost of the soluble polymer (400 € kg^−1^ [74]) minor compared to the savings in biocatalyst production and purification. For the Kollicoat^®^-immobilisate, 3 mg cutinase g_PET_^−1^ was used in this study, a benchmark often used in the literature per batch hydrolysis process to ensure efficient and rapid degradation within 14 h [12, 17]. Accordingly, the catalyst cost was amortised after the first complete hydrolysis cycle, making this immobilisation method already profitable from the second cycle onwards in terms of biocatalyst cost. In addition, yields could be further improved after the fourth cycle by extending the hydrolysis time. The necessary pH adjustments for the separation of the immobilisate involve minimal additional effort and cost, as lowering the pH below 4 is already a required step in the purification of terephthalic acid [47]. This enables efficient enzyme recovery during downstream pH reduction, integrating it seamlessly into the existing purification step without additional steps or resources. Furthermore, in this study, the prior precipitation and separation of the polymer had minimal negative impact on the subsequent terephthalic acid precipitation (> 90% terephthalic acid recovery), demonstrating that the recovery of the immobilised cutinase can be achieved without compromising isolation of the target product. Thus, this method not only simplifies the workflow, but also improves the overall efficiency and sustainability of enzymatic PET degradation.

## Conclusion

Combining high enzymatic activity and recyclability of the enzymes for PET degradation has proven to be challenging as conventional immobilisation methods, such as solid supports, resulted in low activities due to the deteriorated interaction between the catalyst and the macromolecular PET substrate. This study investigated different approaches to overcome this problem. Modifications of existing methods, such as the generation of pCLEAs, suggested that it was possible to establish sufficient interaction between the enzyme and its solid substrate even in immobilised form, but stability and recyclability decreased with more porous cutinase aggregates. Smart polymers, in particular the pH-responsive Kollicoat^®^, showed promising results in the enzymatic degradation of PET and reuse of the cutinase-polymer immobilisate for five efficient cycles of PET hydrolysis on the 1 L-scale, with approximately 80% product yield in the fifth cycle. The immobilisate not only showed high activity and stability, but also simplified downstream processing, as both catalyst and terephthalic acid can be recovered via a pH change. Due to the different pHs required for these two steps, a sequential precipitation of first the enzyme, and then the reaction product is possible, allowing a seamless integration of the biocatalyst recovery with product processing and a simple and elegant process design.

## Supplementary Information

Below is the link to the electronic supplementary material.

Supplementary file1 (PDF 219 KB)

## Data Availability

All data generated and analysed during this study are included in this published article and its supplementary information file. Further data is available from the corresponding author on reasonable request.
