# Supplementary material for: Recycling the recyclers: strategies for the immobilisation of a PET-degrading cutinase
Source: Bioprocess Biosyst Eng. 2025 Feb 2;48(4):605–19. doi: 10.1007/s00449-025-03131-7 (PMC11928388; doi:10.1007/s00449-025-03131-7)
Supplement: Supplementary file 1 — Supplementary file1 (PDF 219 KB) [file 449_2025_3131_MOESM1_ESM.pdf]

Supplementary information

Recycling the recyclers: Strategies for the immobilisation of a PET degrading cutinase

Stefanie Fritzsche[1], Marcus Popp[1], Lukas Spälter[1], Natalie Bonakdar[2], Nicolas Vogel[2], Kathrin Castiglione[1]

1 Institute of Bioprocess Engineering, Department of Chemical and Biological Engineering, Friedrich-Alexander-Universität Erlangen-Nürnberg, Paul-Gordan-Straße 3, 91052 Erlangen, Germany
2 Institute of Particle Technology, Department of Chemical and Biological Engineering, Friedrich-Alexander-Universität Erlangen-Nürnberg, Cauerstraße 4, 91058 Erlangen, Germany

*Corresponding author: kathrin.castiglione@fau.de

Material and Methods

Selection of the precipitating agent To select a suitable precipitant, saturated solutions of AS and polyethylene glycol (PEG, 1000 Da) were used to prepare solutions with varying degree of saturation. After the addition of the enzyme solution to the precipitating agent, the mixtures were stirred at 600 rpm for 1 h. Subsequently, the aggregate-containing suspensions were centrifuged (4 °C, 4,500 g, 30 min), the supernatants were decanted, and the pellets were resuspended in fresh potassium phosphate buffer (100 mM, pH 7.5). Precipitation success at different degrees of saturation was assessed by measuring esterase activity (pNPA assay) after redissolving the resulting pellets in the initial buffer volume.

Characterisation of CLEAs To determine the thermostability of free enzyme and CLEAs at 72 °C, their respective activities were first examined in the pNPA assay. The samples were then incubated at 72 °C and 300 rpm (Thermomixer, Eppendorf SE, Hamburg, Germany). To follow the change in activity over time, the incubations were stopped at regular intervals. The incubated samples, CLEAs or free enzyme, were centrifuged at 4,500 g and 4 °C for 5 min. The supernatants were then separated, the pellets were resuspended again and the pNPA activity was measured for both supernatants and pellets. Using the initial activity of the samples as a reference, the changes in activity over time were evaluated.

Results

ReliZyme^{TM} as solid carrier particles

Table S1 Yields of terephthalic acid equivalents in hydrolysis cycles 1 and 2 using cutinase $ICCG_{DAQI}$ immobilised on ReliZyme^{TM} HA 403/S and EP 403/S particles.

Hydrolysis cycle	HA 403/S	EP 403/S
1	90%	24%
2	1.7%	0.7%

Carrier free immobilisation using CLEAs and pCLEAs

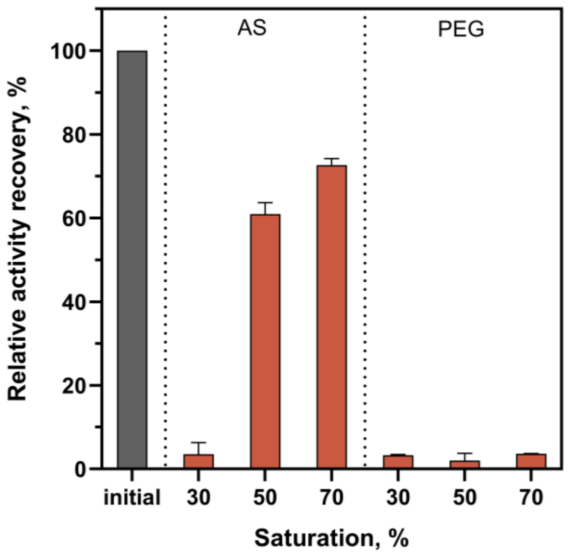

Fig. S1 Relative recovered activity (pNPA assay) after precipitation of a cutinase solution using the precipitating agents AS and PEG at various degrees of saturation. The pellets formed were redissolved in the initial buffer volume after precipitation and the recovered activity was determined in the pNPA assay. The initial activity (100%) was used as a reference (Ref.).

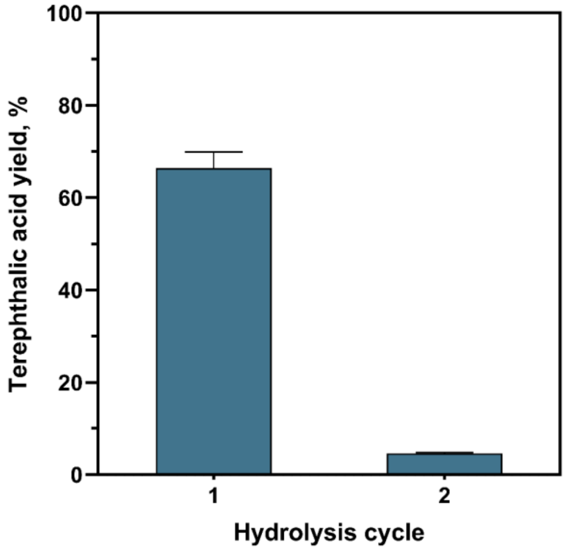

Fig. S2 Terephthalic acid yields in relation to the theoretical maximum after the hydrolysis of textile PET fibres with CLEAs of cutinase $ICCG_{DAQI}$ cross-linked with 10 mM GA. After the first hydrolysis cycle, the cutinase CLEAs were isolated by centrifugation, washed and reused for the hydrolysis of fresh PET fibres.

Immobilisation on stimulus-responsive polymers

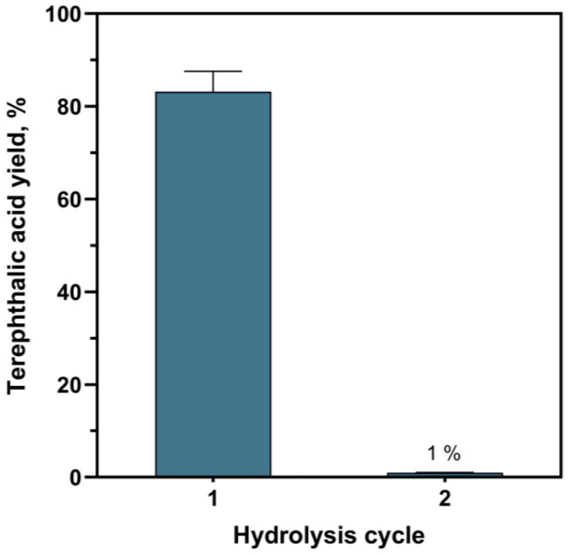

Fig. S3 Terephthalic acid yields in relation to the theoretical maximum after the hydrolysis of textile PET fibres with cutinase $ICCG_{DAQI}$ immobilised on pAA. After the first hydrolysis cycle, the pAA-cutinase immobilisate was precipitated by decreasing the temperature to 4 °C, washed and reused for the hydrolysis of fresh PET fibres.

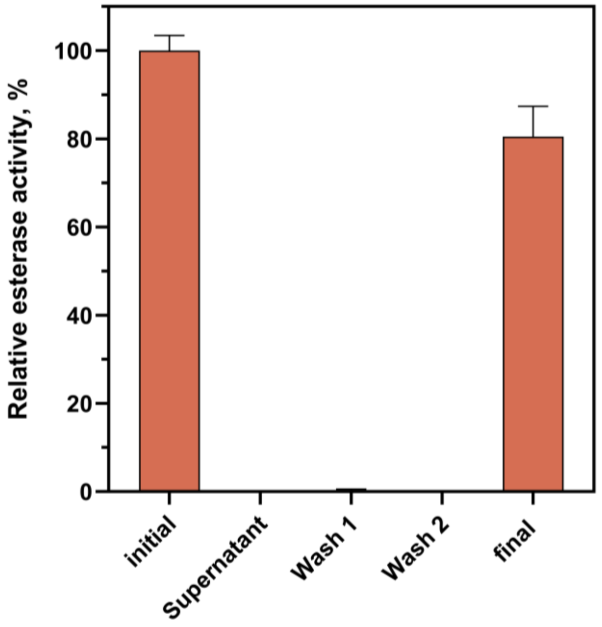

Fig. S4 Relative esterase activity (pNPA assay) of the initial enzyme sample, the supernatant after incubation with Kollicoat®, the washing supernatants with acetate buffer and the final Kollicoat®-enzyme solution. The initial esterase activity refers to the activity of the free enzyme used to load the Kollicoat®. The data points correspond to the mean values ± standard deviation of three esterase measurements.

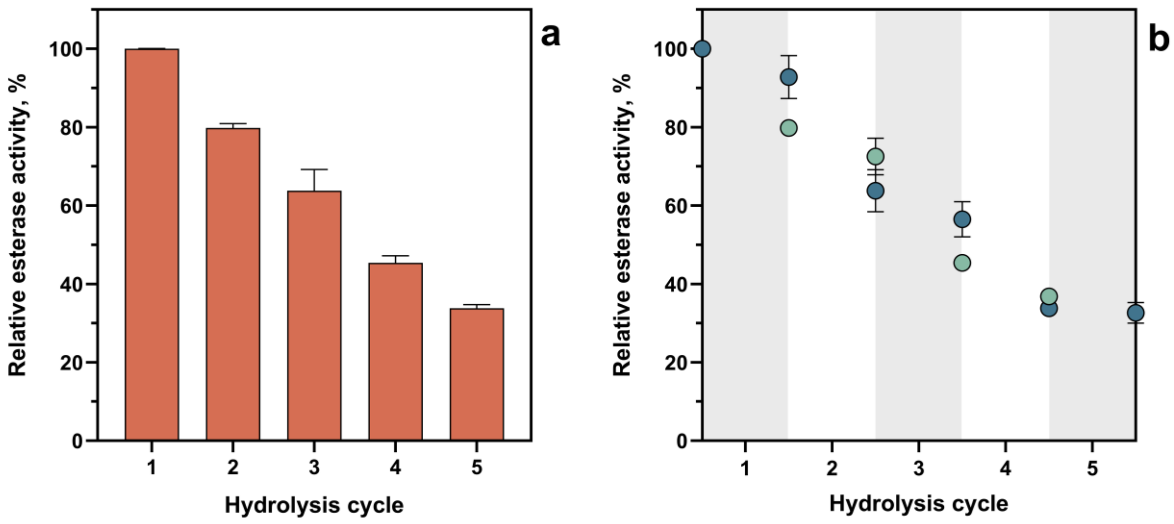

Fig. S5 Relative esterase activity (pNPA assay) of the enzyme immobilised on Kollicoat® compared to its initial activity at the begin of each hydrolysis cycle (**a**) as well as the detailed activity profile before and after each recycling step (**b**). After each cycle, indicated by a change in the background shading and the symbol colors in (**b**), Kollicoat® was precipitated, washed twice and reused for hydrolysis of fresh PET fibres. The initial esterase activity was 81% of the activity of the free enzyme used to prepare the Kollicoat®-cutinase-immobilisate. Each data point represents the mean ± standard deviation of three esterase activity measurements.

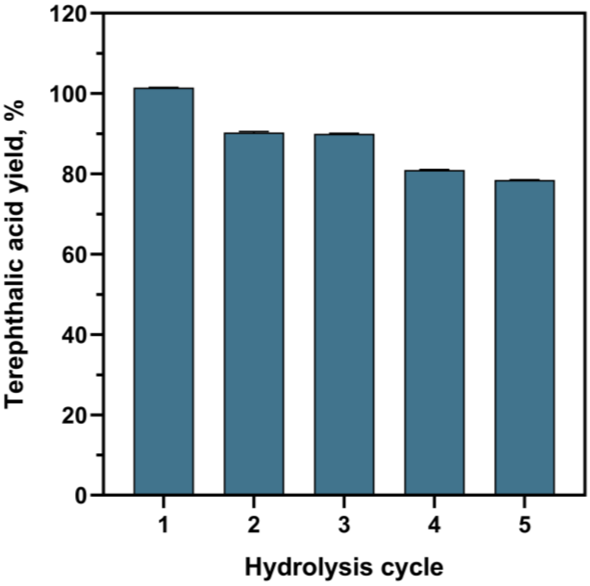

Fig. S6 Terephthalic acid yields in relation to the theoretical maximum after the hydrolysis of textile PET fibres with cutinase ICCG$_{DAQI}$ immobilised via adsorption on Kollicoat® on a 1 L scale. After each of the five hydrolysis cycles, Kollicoat® was precipitated by alteration of the pH, washed and reused for the hydrolysis of fresh PET fibres.

Table S2 Loss of activity of free enzyme and enzyme immobilised on Kollicoat® after 24 h in 0.1 M phosphate buffers with different pH values at 70 °C. In addition, at pH 9, the half-lives $t_{1/2}$ at 70 °C were determined for the free enzyme and the enzyme-Kollicoat®-complexes.

		pH 4	pH 6	pH 8	$t_{1/2}$ at pH 9, d
Loss of activity of	Free enzyme	14 ± 9%	12 ± 4%	9 ± 4%	7.2 ± 0.5
	Immobilised enzyme without EDC	7 ± 4%	9 ± 1%	no loss	8.4 ± 0.3
	Immobilised enzyme with EDC	7 ± 4%	no loss	no loss	10.1 ± 0.5
